# Population Genomics of *Mycobacterium leprae* Reveals a New Genotype in Madagascar and the Comoros

**DOI:** 10.3389/fmicb.2020.00711

**Published:** 2020-05-11

**Authors:** Charlotte Avanzi, Emmanuel Lécorché, Fetra Angelot Rakotomalala, Andrej Benjak, Fahafahantsoa Rapelanoro Rabenja, Lala S. Ramarozatovo, Bertrand Cauchoix, Mala Rakoto-Andrianarivelo, Maria Tió-Coma, Thyago Leal-Calvo, Philippe Busso, Stefanie Boy-Röttger, Aurélie Chauffour, Tahinamandrato Rasamoelina, Aina Andrianarison, Fandresena Sendrasoa, John S. Spencer, Pushpendra Singh, Digambar Ramchandra Dashatwar, Rahul Narang, Jean-Luc Berland, Vincent Jarlier, Claudio G. Salgado, Milton O. Moraes, Annemieke Geluk, Andriamira Randrianantoandro, Emmanuelle Cambau, Stewart T. Cole

**Affiliations:** ^1^Global Health Institute, Ecole Polytechnique Fédérale de Lausanne, Lausanne, Switzerland; ^2^Department of Microbiology, Immunology and Pathology, Mycobacteria Research Laboratories, Colorado State University, Fort Collins, CO, United States; ^3^Department of Medical Parasitology and Infection Biology, Swiss Tropical and Public Health Institute, Basel, Switzerland; ^4^AP-HP, Hôpital Lariboisière, Service de Bactériologie, Centre National de Référence des Mycobactéries et de la Résistance des Mycobactéries aux Antituberculeux - Laboratoire Associé, Paris, France; ^5^Université de Paris, INSERM, IAME UMR1137, Paris, France; ^6^Centre d’Infectiologie Charles Mérieux, Université d’Antananarivo, Antananarivo, Madagascar; ^7^Unité de Soin, de Formations et de Recherche de Dermatologie, University Hospital Joseph Raseta Befelatanana, Antananarivo, Madagascar; ^8^Department of Medecine-Interne, University Hospital Joseph Raseta Befelatanana, Antananarivo, Madagascar; ^9^Fondation Raoul Follereau, Antananarivo, Madagascar; ^10^Department of Infectious Diseases, Leiden University Medical Center, Leiden, Netherlands; ^11^Laboratório de Hanseníase, Instituto Oswaldo Cruz, FIOCRUZ, Rio de Janeiro, Brazil; ^12^Sorbonne Université, INSERM U1135, Centre d’Immunologie et des Maladies Infectieuses, CIMI-Paris, Paris, France; ^13^National Institute of Research in Tribal Health (Indian Council of Medical Research), Jabalpur, India; ^14^Mahatma Gandhi Institute of Medical Sciences, Wardha, India; ^15^Fondation Merieux, Lyon, France; ^16^CIRI, Centre International de Recherche en Infectiologie, Inserm U1111, Lyon, France; ^17^AP-HP, Hôpital Pitié-Salpêtrière, Service de Bactériologie, Centre National de Référence des Mycobactéries et de la résistance des Mycobactéries aux Antituberculeux, Paris, France; ^18^Laboratório de Dermato-Imunologia Universidade Federal do Pará (UFPA), Marituba, Brazil; ^19^Programme National de Lutte Contre la Lèpre, Antananarivo, Madagascar; ^20^Institut Pasteur, Paris, France

**Keywords:** leprosy, *Mycobacterium leprae*, Madagascar, Comoros, genomics, phylogeography

## Abstract

Human settlement of Madagascar traces back to the beginning of the first millennium with the arrival of Austronesians from Southeast Asia, followed by migrations from Africa and the Middle East. Remains of these different cultural, genetic, and linguistic legacies are still present in Madagascar and other islands of the Indian Ocean. The close relationship between human migration and the introduction and spread of infectious diseases, a well-documented phenomenon, is particularly evident for the causative agent of leprosy, *Mycobacterium leprae*. In this study, we used whole-genome sequencing (WGS) and molecular dating to characterize the genetic background and retrace the origin of the *M. leprae* strains circulating in Madagascar (*n* = 30) and the Comoros (*n* = 3), two islands where leprosy is still considered a public health problem and monitored as part of a drug resistance surveillance program. Most *M. leprae* strains (97%) from Madagascar and Comoros belonged to a new genotype as part of branch 1, closely related to single nucleotide polymorphism (SNP) type 1D, named 1D-Malagasy. Other strains belonged to the genotype 1A (3%). We sequenced 39 strains from nine other countries, which, together with previously published genomes, amounted to 242 genomes that were used for molecular dating. Specific SNP markers for the new 1D-Malagasy genotype were used to screen samples from 11 countries and revealed this genotype to be restricted to Madagascar, with the sole exception being a strain from Malawi. The overall analysis thus ruled out a possible introduction of leprosy by the Austronesian settlers and suggests a later origin from East Africa, the Middle East, or South Asia.

## Introduction

Leprosy was declared to be eliminated by the government of Madagascar in 2010, but the disease remains a public health problem, with more than 1,000 new cases reported annually since 2007 (Raharolahy et al., 2016; Suttels and Lenaerts, 2016; WHO, 2019). This is certainly an underestimate. Social exclusion and stigmatization are still common in Madagascar (Raharolahy et al., 2016), where approximately 25% of the new cases manifest with grade 2 disabilities, indicating late diagnosis (Raharolahy et al., 2016; Suttels and Lenaerts, 2016). Despite an efficient leprosy control program, the Comoros are still considered a highly endemic area, with a constant average of 400 new cases documented annually for an average population of 400,000 inhabitants since 2008 and 275 new cases in 2018 (Ortuno-Gutierrez et al., 2019; WHO, 2019). However, the relapse rate is low and only 1.8% of new cases present with grade 2 disability (Hasker et al., 2017; Ortuno-Gutierrez et al., 2019). In the last report of the drug resistance surveillance network, resistance to rifampicin (*rpoB*), dapsone (*folP1*), and quinolones (*gyrA*) was observed only in three primary cases between 2009 and 2015 in Madagascar (Raharolahy et al., 2016; Cambau et al., 2018). No information is currently available for the Comoros.

Leprosy is mainly caused by the non-cultivable pathogen *Mycobacterium leprae* and, to a lesser extent, *Mycobacterium lepromatosis* (Han et al., 2008). The *M. leprae* genotyping system is characterized by four single nucleotide polymorphism (SNP) types (1–4) and 16 SNP subtypes (A–P) divided into eight branches (Monot et al., 2009; Schuenemann et al., 2018). In Madagascar and the Comoros, little is known about the genetic background of circulating *M. leprae* strains. Two epidemiological studies reported the presence of the genotype 1D in Madagascar, but only seven isolates were studied so far (Monot et al., 2009; Reibel et al., 2015). No information is available about the strains currently circulating in the Comoros. Although our inability to cultivate the pathogen *in vitro* has hampered research, recently developed methods allow the sequencing of leprosy bacilli DNA directly from human samples (Schuenemann et al., 2013; Avanzi et al., 2016; Benjak et al., 2018).

Despite Madagascar’s proximity to mainland Africa, the genetic, cultural, and archeological evidence indicate that the Malagasy and the Comorans, the inhabitants of Madagascar and the Comoros, respectively, are of mixed African, Indonesian, and Middle Eastern ancestry (Dewar and Wright, 1993; Burney et al., 2004; Ratsimbaharison and Ellis, 2010; Pierron et al., 2014, 2017). As with several other infectious diseases (Insitute of Medicine, 2010), leprosy also exemplifies the correlation between the dissemination of pathogens and human migrations (Monot et al., 2009). However, establishing the origin of *M. leprae* in an admixed population such as the Malagasy requires comprehensive molecular characterization of the pathogen.

In this investigation, we aimed to characterize the genetic background and predict the origin of the *M. leprae* strains circulating in Madagascar and the Comoros using whole-genome sequencing (WGS).

## Materials and Methods

### Ethics Statement

This study was carried out under the ethical consent of the WHO Global Leprosy Programme surveillance network. All subjects gave written informed consent in accordance with the Declaration of Helsinki.

### Patients and Clinical Samples From Madagascar and the Comoros

A total of 60 skin biopsies from 51 suspected leprosy cases from Madagascar (*n* = 48) and the Comoros (*n* = 3), collected between 2013 and April 2017, were obtained from the Leprosy National Reference Laboratory [Centre d’Infectiologie Charles Mérieux (CICM), Antananarivo, Madagascar] and the Centre National de Référence des Mycobactéries et de la Résistance des Mycobactéries aux Antituberculeux (CNR MyRMA, Paris, France) for WGS characterization (Supplementary Table S1). Additionally, a total of 40 samples were collected after May 2017 at the Centre d’Infectiologie Charles Merieux from 40 suspected or diagnosed leprosy cases for molecular drug-susceptibility testing and genotyping (Supplementary Table S1).

Samples were collected at health facilities by medical staff (Supplementary Table S1). Three DNA extracts (B204, B171, and B191; Supplementary Table S1) from a previous investigation at the Institut Pasteur were also included (Monot et al., 2005).

### Additional Samples for Genotyping Screening

DNA samples were obtained from ongoing or previous studies (Monot et al., 2005; Tió-Coma et al., 2019, 2020) from countries where the *M. leprae* genotype 1D was previously reported—Nepal (*n* = 25), Venezuela (*n* = 15), Bangladesh (*n* = 11), Brazil (*n* = 5), Chad (*n* = 4), Antilles (*n* = 3), India (*n* = 1), and Congo (*n* = 1)—for genotyping by PCR and WGS (Supplementary Tables S2, S3). Additional samples from two Austronesian countries, Philippines (*n* = 18), and Indonesia (*n* = 5), were also included.

### DNA Extractions

The choice of the DNA extraction method for the samples from Madagascar and Comoros was influenced by initial results obtained by Ziehl–Neelsen (ZN) staining and standard PCR previously performed on site (Supplementary Table S1). DNA extraction for initial screening at reference laboratories (CICM and CNR-MyRMA) was carried using the freeze–boiling method as previously described (Woods and Cole, 1989). Around 50–100 mg of all previously characterized PCR- or ZN-positive skin biopsies were re-extracted using the host depletion (HD) method (Avanzi et al., 2016; Benjak et al., 2018). PCR- and ZN-negative biopsies and samples from the second screening step were extracted using the quicker total DNA extraction method (Avanzi et al., 2016; Girma et al., 2018; Supplementary Table S1). For samples collected outside Madagascar and the Comoros, the DNA extraction methods used are described in Supplementary Table S2.

### PCR Amplification of Specific Loci, Molecular Drug Resistance Screening, and Genotyping by PCR Sequencing

Detection of *M. leprae* was performed for the first and second screening (Supplementary Table S1) on all samples, as recommended (WHO SEARO/Department of Control of Neglected Tropical Diseases, 2017), using the *M. leprae*-specific repetitive element (RLEP) primers (Table 1). *M. lepromatosis*-specific PCR (primers LPM244) was performed on all samples that were negative for *M. leprae* (Table 1). To identify genotype-specific SNPs, primers were designed using the Primer3 web tool^1^ and are described in Table 1. For each sample, 5 μl of the starting materials, negative control (water) or positive control (*M. leprae* DNA strain Thai-53, NR19352) was used in 50 μl reactions using the Accustart PCR Mastermix (Quantabio, Beverly, MA, United States), and quality was assessed as previously described (Avanzi et al., 2016). Amplification started with a 3 min initial denaturation step at 94°C, followed by 40 cycles of 30 s denaturation at 94°C, 30 s annealing at 58°C (all PCR primers in Table 1), and extension at 72°C for 30 s; final extension was then at 72°C for 5 min. Amplicon sequencing was done by Genewiz (United Kingdom) or Microsynth (Switzerland).

**TABLE 1:** List of primers used in this study.

Primer name	Target	Purpose	Amplicon size (bp)	Primer sequence (5′–3′)	Nucleic acid modification between strains	References
RLEP-F	RLEP	Detection of *M. leprae* by	450	TGAGGCTTCGTGTGCTTTGC	–	Singh et al., 2015
RLEP-R	RLEP	PCR		ATCTGCGCTAGA AGGTTGCC	–	
RLEPq-F	RLEP	Detection of *M. leprae* by	70	GCAGTATCGTGTTAGTGAA	–	Truman et al., 2008
RLEPq-R	RLEP	quantitative PCR		CGCTAGAAGGTTGCCGTATG	–	
RLEPq-P	RLEP			FAM-TCGATGATCCGGCCGTCGGCG QSY	–	
LPM244-F	*hemN*	Detection of	244	GTTCCTCCACCGACAAACAC	–	Singh et al., 2015
LPM244-R		*M. lepromatosis*		TTCGTGAGGTACCGGTGAAA	–	
rpoB-For	*rpoB*	Amplification of the drug	255	CTGATCAATATCCGTCCGGT	–	WHO SEARO/Department of Control of Neglected Tropical Diseases, 2017
rpoB-Rev		resistance-determining region of *rpoB*		CGACAATGAACCGATCAGAC	–	
folP1-For	*folP1*	Amplification of the drug	254	CTTGATCCTGACGATGCTGT	–	
folp1-Rev		resistance-determining region of *folP1*		CCACCAGACACATCGTTGAC	–	
gyrA-For	*gyrA*	Amplification of the drug	225	ATGGTCTCAAACCGGTACATC	–	
gyrA-Rev		resistance-determining region of *gyrA*		TACCCGGCGAACCGAAATTG	–	
SNP-2921694-F	*ml2446*	Specific to 1D-Malagasy	169	TGTATGAACGCTGGGCAGTA	A1015**G**	This study
SNP-2921694-R		genotype		TCAACCGGGTCACCATAGAT		
SNP-3016895-F	*ml2535*	Specific to 1D genotype	199	GAGCCACTATTTCCCGACAA	C3541**A**	This study
SNP-3016895-R		outside Madagascar		CGTCGTCGATGAGCAAGTAA		

### qPCR of RLEP, an *M. leprae*-Specific Region, Prior to WGS

All DNA samples extracted at EPFL were subjected to quantitative PCR (qPCR) analysis to detect *M. leprae* prior to WGS. The repetitive element RLEP was quantified using TaqMan^®^ PCR amplification as described previously, with minor modifications (Truman et al., 2008). A total of 3 μl of each purified DNA sample, or the positive control (DNA from Thai-53, NR-19352) or the negative control (water), was added to a total PCR reaction volume of 20 μl containing 10 μl of TaqPath ProAmp master mix (Thermo Fisher Scientific, MA, United States), 900 nM of each forward (RLEPq-F) and reverse (RLEPq-R) primer, and 250 nM of the hydrolysis probe (RLEPq-P) (Table 1). The reaction mixtures were prepared in triplicate and amplification started with an initial denaturation step of 10 min at 95°C, followed by 40 cycles of 15 s at 95°C and 1 min 60°C, using the QuantStudio 3 real-time PCR system (Thermo Fisher Scientific, MA, United States). Data analysis was performed with the Thermo Fisher Connect Cloud^2^, and the mean cycle threshold (*C*_*t*_) was calculated for each sample. qPCR values were also used to evaluate the relative amount of *M. leprae* DNA in each sample and provide a GO/NO GO answer prior to WGS.

### Library Preparation and Comparative Genomic Analysis

Up to 1 μg of DNA in 50 μl was fragmented to 300–400 bp by Adaptive Focused Acoustics on a Covaris S2 instrument (Covaris) using the manufacturer’s protocol. After a 1.8 × ratio cleanup using KAPA Pure beads (Roche, Switzerland), DNA library preparation was performed using the KAPA HyperPrep kit (Roche, Switzerland) and the KAPA dual indexes, as described elsewhere (Benjak et al., 2018). After the final amplification step, libraries were quantified using the Qubit dsDNA HS or BR Assay Kit (Thermo Fisher Scientific, MA, United States) and the fragment size assessed on a Fragment Analyzer (Advanced Analytical Technologies, Inc., Ankeny, IA, United States). Finally, libraries were multiplexed and sequenced using single-end reads on Illumina HiSeq 2500 or NextSeq instrument.

Raw reads were processed as described elsewhere (Benjak et al., 2018). The phylogenetic analysis was performed using a concatenated SNP alignment (Supplementary Table S3). Maximum parsimony (MP) trees were constructed in MEGAX (Kumar et al., 2018) with the 72 new genomes from this study (Supplementary Table S2) and 170 previously published genomes (Supplementary Table S4; Honap et al., 2018; Schuenemann et al., 2018) using 500 bootstrap replicates and *M. lepromatosis* as an outgroup. Sites with missing data were partially deleted (arbitrary 80% coverage cutoff), resulting in 4,040 variable sites used for the tree calculation. Dating analyses were done using BEAST2 (v2.5.2) (Volz and Siveroni, 2018), as described previously (Benjak et al., 2018), with 234 genomes (Supplementary Table S5) and an increased chain length from 50 to 100 million. Briefly, the concatenated SNPs for each sample were used for tip dating analysis. Hypermutated strains and highly mutated genes associated with drug resistance were omitted, but sites with missing data as well as constant sites were included in the analysis, as previously described (Benjak et al., 2018). We included only unambiguous constant sites, i.e., loci where the reference base was called in all samples. Indel calling was done using Platypus v0.8.1 followed by manual curation (Rimmer et al., 2014).

### Genome-Wide Comparison

The SNPs and indels of the newly sequenced genomes from Madagascar and Comoros were compared to the 170 previously published genomes (Supplementary Table S3) and the 72 new genomes from this study (Supplementary Table S2). The impact of amino acid substitutions on protein function was predicted using the online tool Provean (Choi and Chan, 2015).

### *M. leprae* Enrichment of Libraries

To obtain enough *M. leprae* coverage, libraries from previously available DNA or DNA extracted using the total DNA extraction method (Supplementary Table S2) were target enriched for the *M*. *leprae* genome using a custom MYbaits Whole Genome Enrichment kit as described by Honap et al. (2018). Approximately 1.5 μg of each DNA library was captured and pooled with another library of a similar *C*_*t*_ prior to enrichment. Hybridization was performed at 65°C for 48 h. Each enrichment was followed by a second amplification step as per the manufacturer’s recommendations.

## Results

### Retrospective PCR Screening and WGS of Strains From Madagascar and the Comoros

**FIGURE 1:**
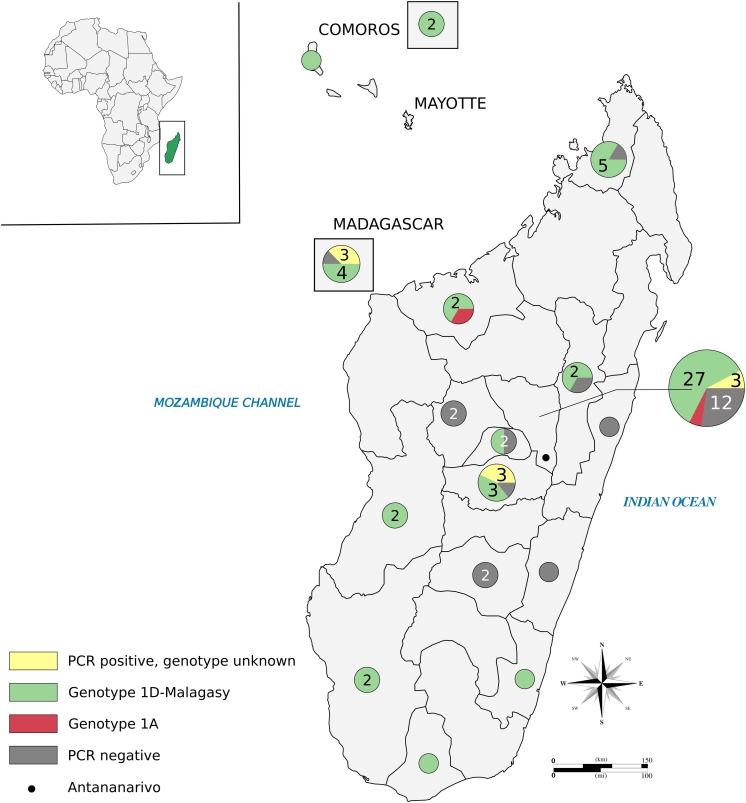
Sampling sites in Madagascar and the Comoros. *Pie charts* indicate the regions where patients originated and are color-coded based on PCR and genotyping results, as indicated in the caption box. *Numbers within circles* represent different patients tested when there is more than one patient. Most of the samples were collected in Antananarivo State. *Boxed circles* refer to the eight patients of unknown location in the island. Data used for the map are available in Supplementary Tables S1, S2 (86 patients). Multiple samples derived from one patient are counted only once. The figure was drawn in Inkscape (Yuan et al., 2016). The map was downloaded from https://www.amcharts.com/svg-maps/ under a free license and modified for the current figure.

Among the 51 patients included retrospectively in this study, 17 were female and 32 were male (two unknown), ranging from 2 to 75 years in age (Supplementary Table S1). They originated from 14 of the 22 regions in Madagascar and the Comoros; their origins are shown in Figure 1 (Supplementary Table S1). Four patients were considered as recurrent cases, and two samples (first and second episodes) were available for only one patient, 02018. Initially, 30 out of 60 samples showed PCR and/or ZN positivity (Supplementary Table S1), for which DNA was re-extracted using the HD method prior to whole-genome quenching characterization. Among the 30 ZN- and PCR-negative samples re-extracted using total DNA extraction, 17 were positive by RLEP PCR (Supplementary Table S1). A second biopsy was available for 11 of 17 positive samples and DNA was re-extracted using the HD method (Supplementary Table S1). The 13 samples negative for *M. leprae* by PCR were also negative for *M. lepromatosis*. Most of the patients with negative PCR and ZN results presented with tuberculoid or paucibacillary leprosy forms, which are characterized by a low amount of bacteria in the skin. Additionally, two negative cases were children and one patient was sampled during a reaction stage. In both cases, the amount of bacteria was also considered low. Finally, one sample was collected for differential diagnosis from a child; the negativity was interpreted as indicating an unrelated disease.

All 41 HD-extracted DNA samples were considered for WGS (Supplementary Table S1). Initial screening showed that efficient WGS (coverage > 5) was achieved in all cases for samples with a qPCR *C*_*t*_ < 28, while only two out of six genomes were recovered in samples with a *C*_*t*_ > 28 (Supplementary Table S1). Md09041 was initially positive by PCR following tDNA extraction, but was negative after HD extraction. For this reason, five samples with a *C*_*t*_ > 28 were not prepared for WGS (Supplementary Table S1). One library failed the quality controls after amplification and was not sequenced. All other DNA extracts (*n* = 35) were sent for library preparation and sequencing (Supplementary Table S2). Three DNA extracts from our 2005 study (Monot et al., 2005) presented *C*_*t*_ values between 17.5 and 28.1 by qPCR (Supplementary Table S1). Libraries were target-enriched using bait capture, but only the sequences of two strains, B191 and B204, met the inclusion criteria of coverage > 5 × (Supplementary Table S1).

Overall, a total of 33 genomes, from 27 patients (*n* = 30) from six regions of Madagascar and the Comoros (*n* = 3), were sequenced with more than 5 × average coverage of non-duplicated reads (Supplementary Tables S2, S6).

### Genome-Wide Analysis of *M. leprae* Strains From Madagascar and the Comoros

#### Genotyping and Phylogeny

**FIGURE 2:**
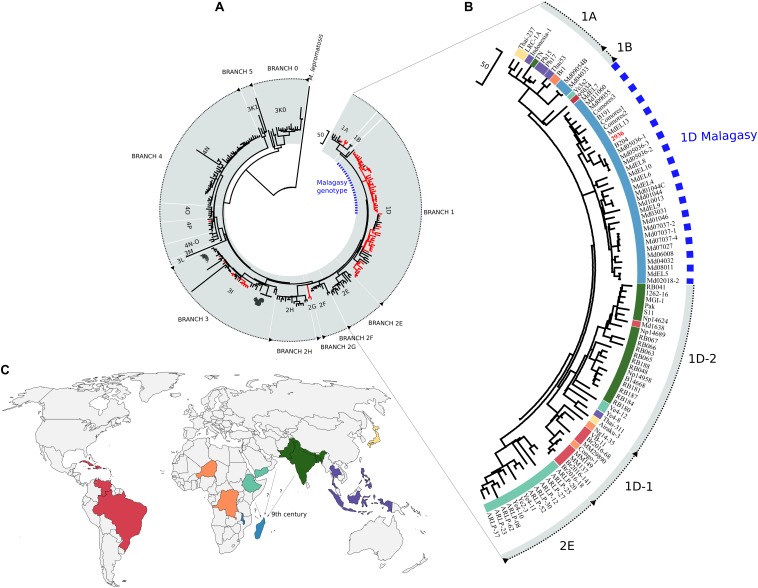
Phylogeography of *Mycobacterium leprae* strains. **(A)** Maximum parsimony tree of 241 genomes of *M. leprae* representing the nine branches and the 16 genotypes. Support values were obtained by bootstrapping 500 replicates. Branch lengths are proportional to nucleotide substitutions. The tree is rooted using *Mycobacterium lepromatosis*. The 1D-Malagasy genotype, discovered in this investigation, is shown in *blue*. Newly sequenced genomes are shown in *red*. **(B)** Zoom into branch 1 (genotypes 1A, 1B, 1D, and the 1D-Malagasy) and 2E of the maximum parsimony tree from **(A)**. The 1D-Malagasy genotype is indicated with the *dotted blue line* and the strain from Malawi in *bold red*. **(C)** Global distribution of the genotypes from the branches 1 and 2E. Genotypes are colored as in **(B)**. Strains from the canonical 1D are found in 12 countries, while the 1D-Malagasy is found only in Madagascar, the Comoros, and Malawi. The *arrows* indicate possible routes of leprosy introduction into Madagascar and Comoros with the estimated time frame.

All the sequenced *M. leprae* strains from Madagascar and the Comoros belonged phylogenetically to branch 1 (Figure 2; Schuenemann et al., 2018). At the SNP subtype level, apart from two SNP subtype 1A, all other strains corresponded to the SNP type 1D (Monot et al., 2009; Benjak et al., 2018). Interestingly, SNP type 1D from Madagascar and the Comoros clustered with strain 2936, previously obtained from Malawi (Benjak et al., 2018), and together these formed a distinct clade that is closely related to the other SNP type 1D strains (Figure 2). The canonical SNP type 1D is composed of two monophyletic groups, including 1D strains from Asia on the one hand and strains from Africa and South America on the other (Figure 2), previously described as 1D-1 and 1D-2 genotypes, respectively (Singh et al., 2014). Branch 1 is now composed of the genotypes 1A, 1B, 1D, and the new 1D-Malagasy genotype.

Of all the 119 new samples from Madagascar (*n* = 30; Supplementary Table S1) and 10 other countries (*n* = 89; Supplementary Table S7) that were either whole-genome-sequenced or PCR-genotyped, the 1D-Malagasy genotype was restricted to Madagascar and the Comoros (30/119). The Malagasy genotype 1D is thus predominant in Madagascar, accounting for 97% of the strains present in 10 of the 14 regions in the country tested (Figure 1).

Aside from the Malagasy samples, 39 new *M. leprae* strains from eight countries, chosen for their proximity to Madagascar or based on the genotyping results previously obtained (Monot et al., 2009) or from this study, were sequenced, representing four different genotypes: 1D, 2G, 3I, and 4P (Supplementary Tables S2–S5). We report here the first whole-genome sequences of two genotype 2G strains, which cluster on a new branch in the phylogeny, falling between branches 2F and 2H (Figure 2).

#### Dating

The most recent common ancestor (MRCA) of all the *M. leprae* strains from branch 1 is estimated to be 2,315 years old [95% highest posterior density (HPD) of 1,903–2,798 ya] and was probably derived from a genotype 2 strain (Supplementary Figures S1, S2). The divergence time of the MRCA of the 1D and 1D-Malagasy strains is 2,270 ya (95% HPD = 1,870–2,744 ya). The divergence time of the MRCA of the 1D-Malagasy strains is 1,132 ya, i.e., the ninth century C.E. (95% HPD = 878–1,417 ya), whereas inside the genotype 1A, the two strains from Madagascar seem to have appeared more recently, around the late 17th century (95% HPD = 175–494 ya; median = 322 ya) (Supplementary Figure S2).

#### Genome-Wide Analysis of the 1D-Malagasy Genotype

From the genome-wide comparison of the 33 new strains from Madagascar and Comoros with 209 other *M. leprae* genomes, 18 polymorphisms were found only in the Malagasy 1D subtype (Supplementary Table S8), including two missense mutations in protein coding sequences. One occurs in *ml0242* (16G > T, Val6Phe), encoding an essential enzyme of the isoprenoid biosynthesis pathway, IspE or 4-diphosphocytidyl-2-C-methyl-D-erythritol kinase. The mutation was predicted to be deleterious for the protein function (PROVEAN score, −4.393). The other mutation was found at the end of *ml2446* (1015 A > G, Asn339Asp) encoding lipoprotein Q, LprQ. This mutation was predicted to be neutral (PROVEAN score, −2.163).

Interestingly, in all 1D-Malagasy strains, a single nucleotide insertion affects the stop codon of *ml1328* (1581156 G > GT; Ter453fs), coding for the proteasome accessory factor A, PafA. In *M. leprae*, as in *M. tuberculosis*, *pafA* is part of a transcriptional unit with *ml1329* (*pafB*) and *ml1330* (*pafC*) (Festa et al., 2007). Only six nucleotides separate *pafA* from *pafB*, while the stop codon of *pafB* overlaps the start codon of *pafC*. In *M. tuberculosis*, all three genes are co-transcribed, but not essential for growth (Festa et al., 2007). The insertion of a T before the *pafA* stop codon is predicted to lead to the production of a protein that is nine amino acids longer and to the loss of the *pafB* start codon.

## Discussion

The African continent is home to multiple *M. leprae* genotypes, and this study brings additional complexity to the picture. In summary, branch 4 strains seem to be restricted to West Africa, whereas branches 2E, 2F, and 2H are present in East Africa, including Ethiopia (2E, 2F, and 2H) and Malawi (2E) (Monot et al., 2009; Benjak et al., 2018). Strains from branches 1 and 2 have been reported in the Congo (Reibel et al., 2015), while branch 3 strains, notably of genotype 3I, have been described in Morocco and Egypt (Monot et al., 2009). The canonical SNP type 1D is found in 12 countries outside Africa (Monot et al., 2009; Benjak et al., 2018). In Africa, a single 1D strain was found in Niger (Benjak et al., 2018), and one was found in the Congo in this study. The new 1D-Malagasy genotype is prevalent in Madagascar and the Comoros. The only other genome available from Southeast Africa, Malawi (Benjak et al., 2018), also belongs to the 1D-Malagasy genotype. Monot et al. (2009) reported the presence of the genotypes 1D and 2E in Malawi using the standard genotyping system, but more screening will be necessary to establish the frequency of the 1D-Malagasy genotype in the country and elsewhere on the continent. The most ancestral lineage of *M. leprae*, branch 0 (Schuenemann et al., 2018), has not been reported in Africa. Altogether, these data suggest that human migrations have mainly contributed to the introduction of different *M. leprae* genotypes from elsewhere.

The first record of humans in Madagascar is from the beginning of the first millennium with the arrival of Austronesians from the Sunda Islands, ∼4,000 mi. to the East of Madagascar (Dewar and Wright, 1993; Pierron et al., 2014, 2017). The permanent residential settlement of inhabitants in Madagascar is estimated at 700 C.E., with a colonization wave of Austronesians from East Asia and by the Bantu from East Africa (Pierron et al., 2014; Crowther et al., 2016). This also coincides with the entry of East Africa into the Indian Ocean trade, connecting the continent with Asia and the Middle East around 800 C.E. (Seland, 2013; Lawler, 2014; Crowther et al., 2016). An additional migration wave was observed between 1,000 and 1,500 C.E., with individuals of Austronesian, Bantu, and Middle Eastern origins (Pierron et al., 2014). The *M. leprae* SNP type 1A, found at a very low frequency (3%) in Madagascar, is mostly reported in Southeast Asia (Philippines and Indonesia, 90 and 60%, respectively), North India and Nepal (2%), Thailand (one strain), Korea (50%), and Bangladesh (50%) (Monot et al., 2009).

Our data suggest that the subtype 1A was introduced into Madagascar and Comoros after East Africa entered the Indian trade route around 800 C.E., or when the East India Company began the slave trade with Madagascar in the 17th century (Thomas, 2014). The MRCA of the 1D-Malagasy genotype was likely a SNP type 2 strain, which was circulating in medieval Europe and is currently prevalent in East Africa and the Middle East. The MRCAs of the canonical 1D and the 1D-Malagasy strains further suggest an introduction of the 1D-Malagasy genotype between the third century B.C.E and ninth century C.E. The 1D-Malagasy genotype was found in 10 regions in Madagascar and in three different countries (Madagascar, Comoros, and Malawi). The 1D-Malagasy clade is composed of several monophyletic groups. We anticipate that most of the genetic diversity for this genotype has been captured during this investigation, suggesting that the strain was introduced into Madagascar and the Comoros no earlier than the ninth century C.E. Besides, the estimates of our model overlap with the previous estimates reported by Schuenemann et al. (2018), with the MRCA of branch 1 being 2,248 years old vs. 2,315 years old in our study. Altogether, these data rule out Austronesian migrations as the origin of the 1D-Malagasy *M. leprae* genotype found in Madagascar and, rather, point to an introduction from East Africa, the Middle East, or South Asia around the time of the Indian Ocean trade (Lawler, 2014). However, the exact origin of the 1D-Malagasy genotype is difficult to pinpoint due to the near-complete absence of genomic information from the neighboring countries or countries where the canonical 1D genotype was previously reported, like India and the Middle East (Monot et al., 2009; Lavania et al., 2015). The sole exception is strain 2936 from Malawi, which is highly related to four isolates from Madagascar (Figure 2 and Supplementary Figures S2, S3). Furthermore, yet another argument against the Austronesian origin of leprosy in Madagascar is the relatively young age of the 1A genotype, which is the prevalent genotype in Southeast Asia (Phetsuksiri et al., 2012). Nevertheless, additional investigation using the specific 1D-Malagasy marker on the islands, in surrounding countries, and those where genotype 1D occurs should help to retrace the exact origin of the 1D-Malagasy genotype and obtain a full picture of the strain’s diversity.

There is a strikingly low strain diversity in Madagascar and the Comoros compared to other islands such as New Caledonia or the Antilles (Monot et al., 2009), where several different genotypes have been observed. This is consistent with the relative isolation of the Malagasy population and the lower immigration into Madagascar in the last centuries compared to other islands or oceanic regions located on major routes of trade or migration.

## Data Availability Statement

The datasets generated for this study can be found in the NCBI Sequence Read Archive (SRA) under accession number PRJNA592722.

## Ethics Statement

This study was carried out under the ethical consent of the WHO Global Leprosy Programme surveillance network. All subjects gave written informed consent in accordance with the Declaration of Helsinki.

## Author Contributions

CA, SC, MR-A, J-LB, and EC designed the study. LR, FRR, BC, AC DD, RN, AA, FS, and AR collected the samples for this study. JS, MM, AG, CS, AA, and VJ collected the samples as part of other ongoing studies. CA, EL, FAR, PS, MT-C, TL-C, and TR performed DNA extraction, molecular screening, and WGS. SB-R and PB, and PS performed PCR sequencing. CA, AB, MR-A, and SC processed the experimental data. CA and AB performed the computational analysis. CA, AB, SC, EL, and EC drafted the manuscript. All authors discussed the results and commented on the manuscript.

## Conflict of Interest

The authors declare that the research was conducted in the absence of any commercial or financial relationships that could be construed as a potential conflict of interest.

## Footnotes

1 http://primer3.sourceforge.net

2 https://www.thermofisher.com/ch/en/home/cloud.html
