# Supplementary material for: Population Genomics of Mycobacterium leprae Reveals a New Genotype in Madagascar and the Comoros
Source: Front Microbiol. 2020 May 11;11:711. doi: 10.3389/fmicb.2020.00711 (PMC7233131; doi:10.3389/fmicb.2020.00711)
Supplement: Supplementary file 1 [file Data_Sheet_1.docx]

Supplementary Materials

# Supplementary Figures and Tables

## Supplementary Tables

**Supplementary Table 1**: Dataset of samples obtained from Madagascar and Comoros from the initial screening in references laboratories in Antananarivo (CICM) and Paris (CNR Mycobactérie); retrospective screening (first screening) and after May 2017 (second screening), patient information, collection sites, molecular, histopathological and genotyping results – WT: sequence wild-type, NA: data not available; ND: experiment not performed; IPM: Institut Pasteur of Madagascar; HJRB: Hopitâl Joseph Raseta Befelatanana; NEG: negative, POS: positive, HD: host depletion DNA extraction, tDNA: total DNA extraction, ZN: Ziehl-Neelsen, tDNA/HD: total DNA extraction was performed on the first sample and if PCR was positive, DNA extraction was performed using HD method on a second biopsy.

**Supplementary Table 2**: List of the 72 newly sequenced genomes, sequencing information, dating, DNA extraction methods used and origin.

**Supplementary Table 3**: List of the 170 genomes published, origin and dating used for comparative genomic in this study.

**Supplementary Table 4**: SNP table for the 243 genomes - SNP calling was done using VarScan v2.3.9. The software SnpEff v4.2 was applied to estimate the effect of genomic variation.

**Supplementary Table 5**: Dataset used for molecular dating with BEAST in a zipped xml file

**Supplementary Table 6**: Patient information of the 39 newly sequenced genomes from outside Madagascar – NA: information not available

**Supplementary Table 7**: Genotyping results obtained by PCR or whole genome sequencing of strains outside Madagascar – ND: experiment not done; 3016895: *M. leprae* genome coordinate (TN reference) of the informative site for the canonical 1D (Table 1); 2921694: *M. leprae* genome coordinate (TN reference) of the informative site for the 1D-Malagasy genotype.

**Supplementary Table 8**: Specific variants restricted to the 1D-Malagasy genotype compared to the whole dataset of *M. leprae* genomes.

## Supplementary Figures



**Supplementary Figure 1.** Bayesian phylogenetic tree of 235 genomes of *M. leprae* calculated with BEAST 2.4.4. Hypermutated strains 85054, Amami, S15, Br14-3, Br2016-15, Zensho-4, Zensho-5 and Zensho-9 (Benjak et al., 2018) with mutations in the *nth* gene were excluded from the analysis. The tree is drawn to scale, with branch lengths representing years of age. Orange horizontal bars show the 95% Highest Posterior Density range of the age for each node.





**Supplementary Figure 2.** Zoom into the Branch 1 (genotypes 1A, 1B, 1D and the 1D-Malagasy) and 2E of the Bayesian phylogenetic tree from the Supplementary Figure 1.
